# Cysteine- and glycine-rich protein 1 predicts prognosis and therapy response in patients with acute myeloid leukemia

**DOI:** 10.1007/s10238-023-01269-w

**Published:** 2024-03-28

**Authors:** Qianqian Hao, Yu Liu, Yajun Liu, Luyao Shi, Yufei Chen, Lu Yang, Zhongxing Jiang, Yanfang Liu, Chong Wang, Shujuan Wang, Ling Sun

**Affiliations:** 1 Department of Hematology, The First Affiliated Hospital of Zhengzhou University, No. 1 Jianshe East Road, Erqi District, Zhengzhou, 450052 China; 2 Department of Orthopaedics, Warren Alpert Medical School/Rhode Island Hospital, Brown University, Rhode Island, USA

**Keywords:** Acute myeloid leukemia, Adult, Biomarker, Prognosis, *CSRP1*

## Abstract

Acute myeloid leukemia (AML) is a heterogeneous disease with a poor prognosis. The current risk stratification system is essential but remains insufficient to select the best schedules. Cysteine-rich protein 1 (*CSRP1*) is a member of the CSRP family and associated with poor clinicopathological features in many tumors. This study aimed to explore the clinical significance and molecular mechanisms of cysteine- and glycine-rich protein 1 (*CSRP1*) in AML. RT-qPCR was used to detect the relative expression of *CSRP1* in our clinical cohort. Functional enrichment analysis of *CSRP1*-related differentially expressed genes was carried out by GO/KEGG enrichment analysis, immune cell infiltration analysis, and protein–protein interaction (PPI) network. The OncoPredict algorithm was implemented to explore correlations between *CSRP1* and drug resistance. *CSRP1* was highly expressed in AML compared with normal samples. High *CSRP1* expression was an independent poor prognostic factor. Functional enrichment analysis showed neutrophil activation and apoptosis were associated with *CSRP1*. In the PPI network, 19 genes were present in the most significant module, and 9 of them were correlated with AML prognosis. The high *CSRP1* patients showed higher sensitivity to 5-fluorouracil, gemcitabine, rapamycin, cisplatin and lower sensitivity to fludarabine. *CSRP1* may serve as a potential prognostic marker and a therapeutic target for AML in the future.

**Supplementary Information:**

The online version contains supplementary material available at 10.1007/s10238-023-01269-w.

## Introduction

Acute myeloid leukemia (AML) is a heterogeneous and highly aggressive hematologic malignancy [1]. It is the most prevalent form of acute leukemia in adults, with an annual incidence of 4.3/100,000 and an increasing incidence risk with older age [2]. Existing standard treatments include intensive chemotherapy and allogeneic hematopoietic stem cell transplantation (HSCT). Since 2017, the U.S. Food and Drug Administration (FDA) has approved eight new drugs for the treatment of AML, including the FLT3 inhibitors midostaurin and gicitinib, the IDH inhibitors efusitinib and enalcitinib, the anti-CD33 monoclonal antibody gemtuzumab ozogamicin, CPX351, the hedgehog pathway inhibitor glasdegib, and BCL-2 inhibitor venetoclax. Targeted therapies are more effective and less toxic than the conventional chemotherapy [3]. Despite significant advances in these new therapies, primary and secondary resistance remains a major obstacle in treating AML. Therefore, pre-evaluation of the likelihood of resistance is vital for treatment options [4, 5]. Allogeneic HSCT has become an effective tool for AML cure by preventing AML relapse through high-dose chemotherapy and graft-versus-leukemia effects. However, how to screen the population for transplantation is still a challenge in AML treatment. According to the current cytogenetic risk stratification, patients are classified into three risk groups (favorable group, intermediate group, and adverse group). However, patients classified as favorable or intermediate group still have high relapse and drug resistance rates, suggesting that the current risk stratification system is still inadequate. Therefore, the creation of novel and trustworthy prognostic biomarkers for AML is urgently required.

Cysteine-rich protein 1 (*CSRP1*) is a member of the CSRP family. This gene family contains a group of LIM domain proteins, which are proposed to be involved in regulatory processes essential for development and cellular differentiation. *CSRP1* is located on human chromosome 1q32.1 [6]. The LIM protein, CRP1, is a general marker for smooth muscle lineages [7]. CRP1 localizes to the nucleus and the cytoplasm with different functions depending on its location. When CRP1 is in the nucleus, it regulates interactions between transcription factors and promotes the upregulation of smooth muscle-specific genes [8]. When CRP1 is in the cytoplasm, it localizes to the adhesion patch and the actin cytoskeleton to regulate actin filament bundles [9].

Only a few reports have been made about *CSRP1*'s connection to cancer thus far. *CSRP1* is associated with poor clinicopathological features in adrenocortical carcinoma (ACC) [10]. Hepatocellular carcinoma (HCC) causes abnormal methylation to inactivate *CSRP1*, which may be a key biomarker for cancer [11]. *CSRP1* was used to forecast when benign prostatic hyperplasia will turn into prostate cancer and may have an effect on disease-free survival [12]. In addition, *CSRP1* is also associated with colorectal cancer [13] and breast cancer [14].

There has been no research to investigate the expression profile and function of *CSRP1* in AML. In this study, we examined the database and our cohort to investigate the expression of *CSRP1* in AML and its prognostic significance. We also studied the differentially expressed genes related to *CSRP1* expression and explored their potential roles in AML through GO and KEGG enrichment, immune infiltration, protein interaction analysis, and drug sensitivity analysis.

## Materials and methods

### Patients and treatment regimen

Bone marrow samples from 224 newly diagnosed AML patients and 23 healthy individuals were obtained at the First Affiliated Hospital of Zhengzhou University between February 2017 and July 2020. We named our cohort as ZZU cohort. The inclusion criteria included: 1) patients were diagnosed as AML in our hospital; 2) patients were ≥ 14 years old; and 3) patients accepted at least one course of chemotherapy in our hospital. The exclusion criteria were patients who didn’t treat or treat elsewhere and patients with acute promyelocytic leukemia. Patient clinical information, such as age, gender, risk stratification, FAB classification, gene mutations, and other basic information, was gathered from electronic medical records. All data were collected independently by two researchers who received training to reliably complete all assessments. Details of treatment regimens are reported [15, 16]. Induction chemotherapy regimens include IA and DA regimens: standard-dose cytarabine (Ara-C) 100–200 mg·m^−2^·d^−1^ × 7 d combined with idarubicin 10–12 mg·m^−2^·d^−1^ × 3d or daunorubicin 60 mg·m^−2^·d^−1^ × 3d. The treatment plan after remission is high-dose Ara-C (2–3 g/m^2^, once every 12 h, 3d). Those who have not undergone HSCT share four courses; those who receive HSCT share two courses, followed by HSCT. For elderly patients over 60 years old or patients who cannot tolerate intensive chemotherapy, they received chemotherapy with demethylating drugs ± CAG ± venetoclax regimen until progression. In total, 38 subjects received an allogeneic HSCT. Subjects were followed up until death, loss to follow-up or December 2022. The follow‐up was conducted through in‐clinic follow‐up and telephone calls. Patients were classified into low *CSRP1* group and high *CSRP1* group according to the expression of *CSRP1* at diagnosis. The clinical significance of *CSRP1* in the AML cohort was analyzed retrospectively. Complete remission, relapse, risk stratification, overall survival (OS), and relapse-free survival (RFS) were defined as described according to the 2017 ELN recommendations from an international expert panel [17]. The study was approved by the Ethics Committee of the First Affiliated Hospital of Zhengzhou University, and written informed consent was exempted by the Ethics Committee of the First Affiliated Hospital of Zhengzhou University.

### RNA extraction, cDNA synthesis and RT-qPCR

Bone marrow mononuclear cells were obtained via density gradient centrifugation. Total RNA was extracted using TRIzol Reagent (Invitrogen, Carlsbad, CA, USA). The cDNA was synthesized using a High Capacity cDNA Reverse Transcription Kit (Applied Biosystems, Foster City, CA, USA) (18). *CSRP1* transcript levels were detected by the Taqman method using real-time quantitative polymerase chain reaction (RT-qPCR) as previously described [18]. Serial dilutions of plasmids expressing *CSRP1* and *ABL1* were amplified to construct standard quantification curves [19]. The *CSRP1* and *ABL1* copy numbers were calculated from standard curves. As previously described, the *CSRP1* transcript levels were calculated as the ratio of the *CSRP1* copy number/ABL1 copy number [20]. The primers and probe sequences are shown in Table S1.

### Data acquisition

We obtained clinical information and gene expression profiles for AML patients from three independent sources. The first source is Tyner's study, which included 405 AML patients (hereafter referred to as the Beat-AML dataset) [21]. The second source is the TCGA database, which provides RNA-seq expression data and detailed clinical data on 173 AML patients (hereafter referred to as the TCGA-LAML dataset) [22]. The third source is the GSE12417 from the GEO database (https://www.ncbi.nlm.nih.gov/geo/; hereafter referred to as the GSE12417 dataset) [23]. The AML samples' Level 3 HTSeq-FPKM and HTSeq-Count data were downloaded from the TCGA website (https://portal.gdc.cancer.gov/repository) for further investigation.

### Survival analyses

We utilized the pROC package to determine the optimal cutoff value for each gene and divided AML patients into two subgroups: the “high-expression” and “low-expression” groups according to the cutoff value of the gene. Then we performed Kaplan–Meier survival analysis to investigate the prognostic value of gene expression using the survival package.

### Differentially expressed gene (DEG) analysis

The DESeq2 R package was used to compare the expression data of low and high expression of *CSRP1* (cutoff value of 50%) in AML samples to identify DEGs [24]. Heatmap analysis of the top 20 DEGs was performed.

### Functional enrichment analysis of DEGs

Functional enrichment analysis was performed on DEGs with |logFC| > 0.5 and padj < 0.05. The ClusteProfiler package [25] in R was used to implement Gene Ontology (GO) functional analysis, which included cellular component (CC), molecular function (MF), and biological process (BP) analysis, as well as Kyoto Encyclopedia of Genes and Genomes (KEGG) pathway analysis.

### Analysis of immune infiltration using single-sample gene set enrichment analysis (ssGSEA)

*CSRP1* immune infiltration analysis was carried out using ssGSEA utilizing the GSVA package [26] in R. As previously stated [27], 24 kinds of infiltrating immune cells were obtained. The Spearman correction was used to examine the relationship between *CSRP1* and the enrichment scores of 24 different kinds of immune cells. The Wilcoxon rank-sum test was used to examine the enrichment scores of the groups with high and low *CSRP1* expression.

### The protein–protein interaction (PPI) network

The Search Tool for the Retrieval of Interacting Genes (STRING) database (http://string-db.org) was used to predict the DEG PPI network [28]. The cutoff criterion was set at an interaction score level of 0.4. The PPI network was mapped with Cytoscape (version 3.6.1) [29], and the most critical modules in the PPI network were identified with MCODE (version 1.5.1) [30]. The following were the selection criteria: MCODE scores greater than 5, degree cutoff = 2, node score cutoff = 0.2, Max depth = 100, and *k*-score = 2.

### Generation and prediction of prognostic models

A nomogram was developed using the rms R package (version 6.2–0) to individualize the prediction of overall survival (OS) in AML patients, which comprised key clinical features and calibration plots. The calibration curves were visually assessed by plotting the nomogram-predicted probabilities against the observed rates, with the 45 line representing the best predictive values. All statistical tests were two-tailed, and the statistical significance threshold was set at 0.05.

### Chemotherapy response prediction

The oncoPredict package of R was used to forecast medication sensitivity to 5-fluorouracil, gemcitabine, rapamycin, cisplatin, and fludarabine with the goal of enhancing tailored therapy. Accordingly, drug sensitivity values (measured by AUC, the area under the concentration–response curve) were estimated followed by a comparison of the values between the high and low *CSRP1* groups. High AUC means low sensitivity.

### Statistical investigation

R (3.6.3) was used to perform and show all statistical analyses and figures. Wilcoxon rank-sum test was used to evaluate *CSRP1* expression in unpaired samples, while Wilcoxon signed-rank test was utilized in paired samples. Quantitative data are presented as means ± SD (standard deviations) or medians with interquartile ranges. To assess the connection between clinical/cytogenetic features and *CSRP1* expression, the Kruskal–Wallis test, Wilcoxon signed-rank test, and logistic regression analysis were performed. The prognostic variables were evaluated using Cox regression analysis and the Kaplan–Meier technique. The cutoff of *CSRP1* was calculated by X-tile. There were few missing data on the key variables in our main analysis. Participants who were lost to follow-up were deemed alive after loss to follow-up. Multivariate analyses were conducted, controlling for confounders.

## Results

### Overexpression of CSRP1 in AML and expression of CSRP1 in pan-cancers

**Fig.1:**
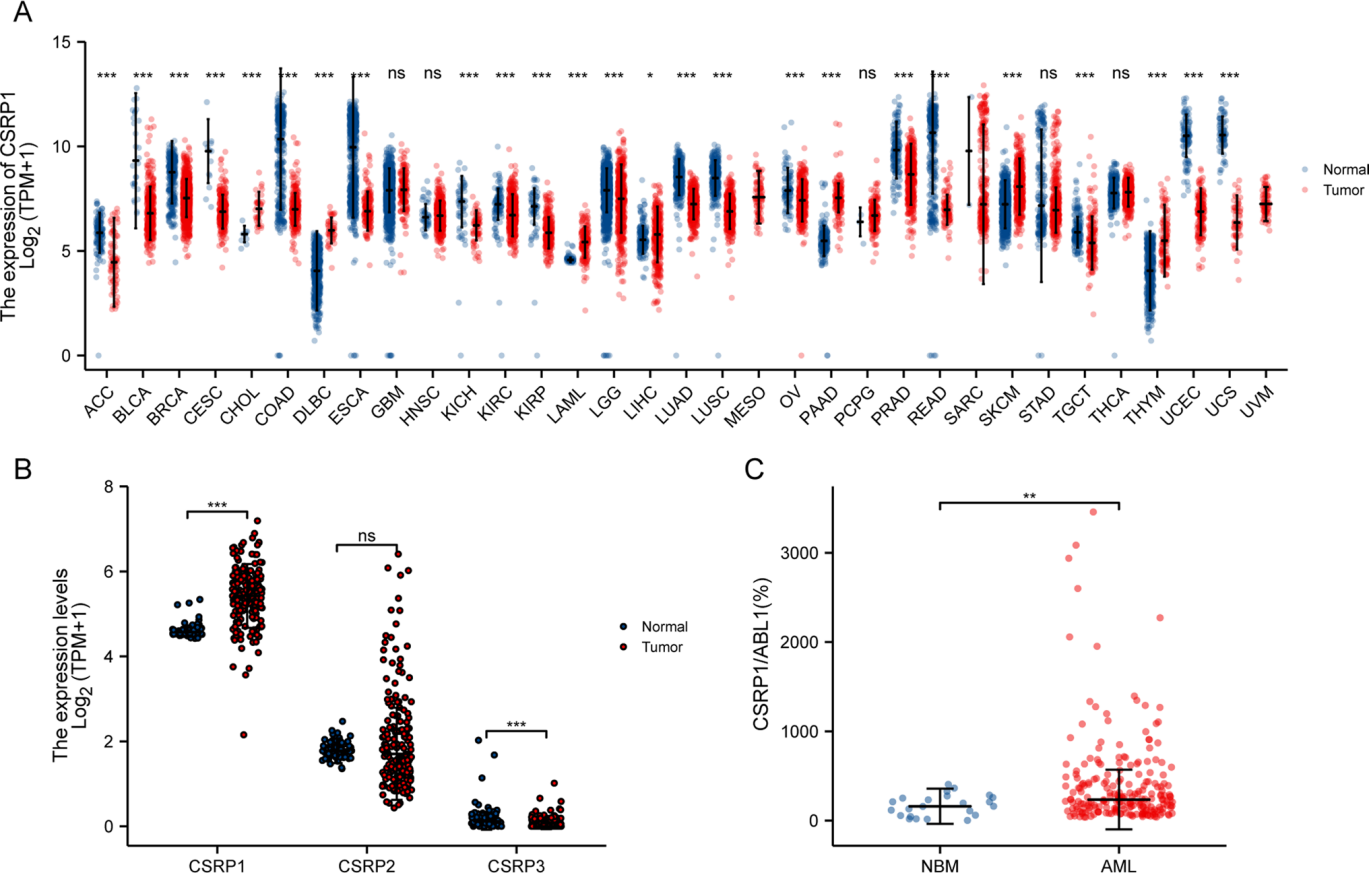
AML samples showed a higher expression of *CSRP1* compared to normal samples. **A** Expression levels of *CSRP1* in paired samples of normal and tumor patients in different cancers. **B** Comparisons of gene expressions among different CSRP family for normal and AML samples. **C** AML samples from the ZZU cohort showed a significant increase in *CSRP1* expression compared to normal bone marrow samples. *, *P* < 0.05; **, *P* < 0.01; ***, *P* < 0.001

RNA-seq data were obtained in the TCGA and GTEx databases. By comparing the expression of *CSRP1* in normal samples in the GTEX database and corresponding tumor samples in the TCGA database, *CSRP1* was found to be significantly overexpressed in 7 cancers, including acute myeloid leukemia (LAML), and underexpressed in 18 cancers (Fig. 1A). For the *CSRP* gene family, *CSRP1* was significantly overexpressed in AML, while *CSRP3* was significantly reduced in AML compared to healthy controls (Fig. 1B). Furthermore, by comparing the expression of *CSRP1* in 23 normal bone marrow (NBM) and 224 newly diagnosed AML patients in the ZZU cohort, we confirmed that *CSRP1* was overexpressed in AML (Fig. 1C).

### Association between CSRP1 expression and clinical features

**Fig.2:**
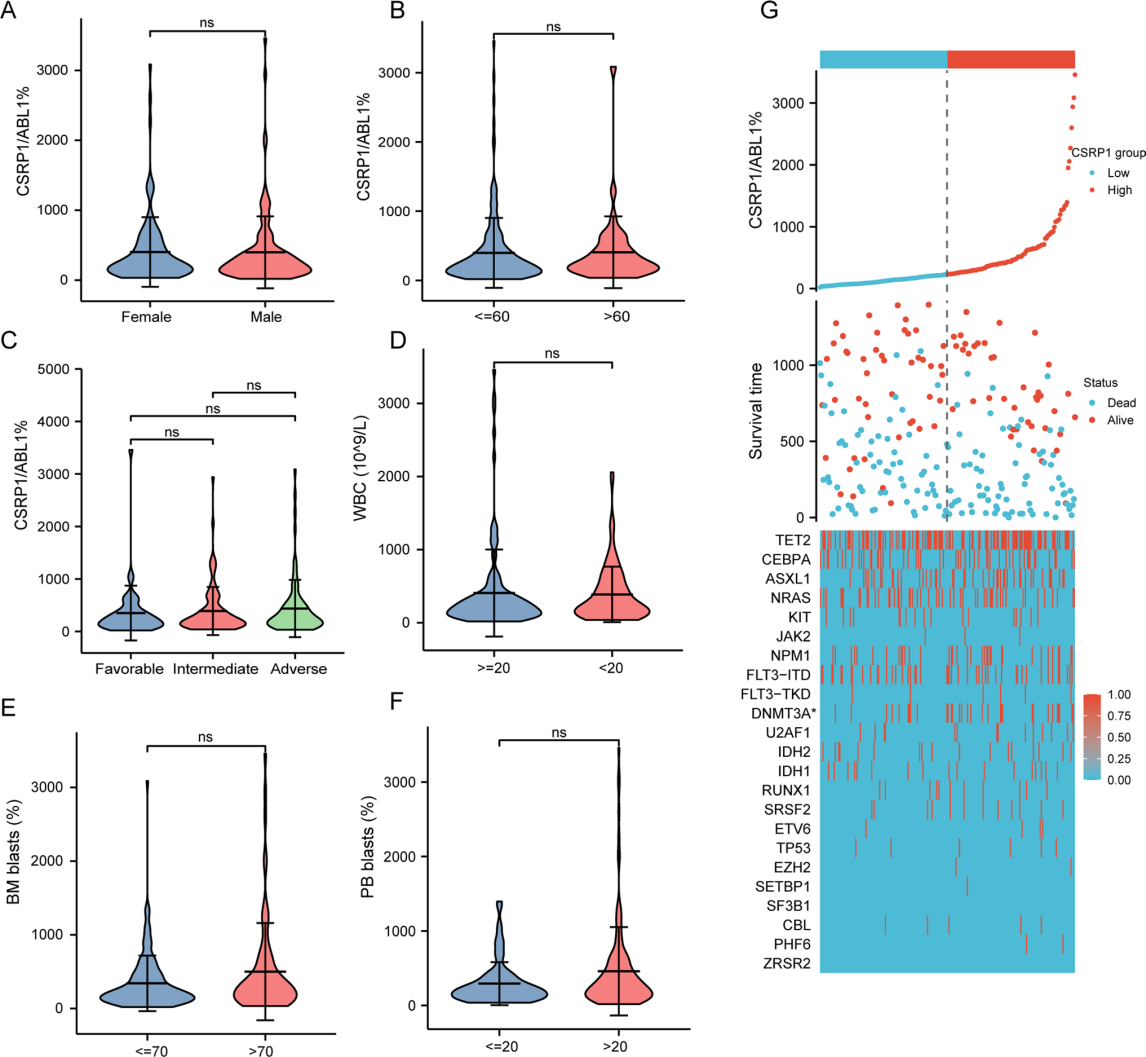
Association between *CSRP1* expression and clinical features in the ZZU cohort. **A**–**F** Comparisons of *CSRP1* expression in AML patients based on gender **A**, age **B**, cytogenetics risk **C**, WBC **D**, BM-blasts **E**, PB-blasts **F**. **G** Association among *CSRP1* expression, survival status and common gene mutations in AML. ns: *P* > 0.05; *, *P* < 0.05

The main clinical characteristics of AML in the ZZU cohort are shown in Table 1. A total of 224 AML patients were included in the prognostic analysis. The median follow-up time was 529 days. Up to the follow-up time, 138 patients died. The AML patients were divided into the low *CSRP1* group (109 cases) and the high *CSRP1* group (115 cases) according to the cut-off *CSRP1* expression (225%). The high *CSRP1* group had higher BM-blasts and more *DNMT3A* mutations than the low *CSRP1* group (*P* < 0.05, Table 1 and Fig. 5G). We also compared the expression of *CSRP1* in AML patients by the other characteristics. The expression levels of *CSRP1* showed no significant difference in patients with different gender, age, WBC, cytogenetic risk group, BM-blasts, and PB-blasts (*P* > 0.05; Fig. 2A–F).

**Table 1:** Association between *CSRP1* expression and clinical features in the ZZU cohort

Characteristic	low *CSRP1* group	high *CSRP1* group	*P*
*n*	109	115	
Age, mean ± SD	44.81 ± 16.56	44.74 ± 15.61	0.975
WBC, median (IQR)	25.05 (7.6, 62.18)	23.7 (6.19, 67.19)	0.625
PB-blasts, median (IQR)	38.5 (0.84, 80)	49.5 (8, 85)	0.089
BM-blasts, median (IQR)	46.6 (0.83, 72.3)	59.6 (24.4, 84.6)	0.021
Risk, *n* (%)			0.651
Favorable	24 (22%)	23 (20%)	
Intermediate	47 (43.1%)	45 (39.1%)	
Adverse	38 (34.9%)	47 (40.9%)	
*MLL*, *n* (%)	1 (0.9%)	5 (4.7%)	0.116
*ETO*, *n* (%)	14 (13%)	11 (10.3%)	0.689
*CBFβ-MYH11*, *n* (%)	8 (7.3%)	4 (3.8%)	0.400
*TET2*, *n* (%)	50 (45.9%)	58 (50.4%)	0.583
*CEBPA*, *n* (%)			0.293
Biallelic mutated	1 (0.9%)	4 (3.5%)	
Others	5 (4.6%)	9 (7.8%)	
*ASXL1*, *n* (%)	19 (17.4%)	27 (23.5%)	0.340
*NRAS*, *n* (%)	27 (24.8%)	23 (20%)	0.486
*KIT*, *n* (%)	10 (9.2%)	7 (6.1%)	0.535
*JAK2*, *n* (%)	1 (0.9%)	2 (1.7%)	1.000
*NPM1*, *n* (%)	13 (11.9%)	22 (19.1%)	0.194
*FLT3*, *n* (%)	21 (19.3%)	29 (25.2%)	0.364
*FLT3-ITD*, *n* (%)	21 (19.3%)	25 (21.7%)	0.770
*FLT3-TKD*, *n* (%)	2 (1.8%)	4 (3.5%)	0.684
*DNMT3A*, *n* (%)	9 (8.3%)	22 (19.1%)	0.031
*U2AF1*, *n* (%)	4 (3.7%)	10 (8.7%)	0.202
*IDH2*, *n* (%)	9 (8.3%)	8 (7%)	0.909
*IDH1*, *n* (%)	8 (7.3%)	9 (7.8%)	1.000
*RUNX1*, *n* (%)	5 (4.6%)	6 (5.2%)	1.000
*SRSF2*, *n* (%)	4 (3.7%)	8 (7%)	0.427
*ETV6*, *n* (%)	1 (0.9%)	3 (2.6%)	0.622
*TP53*, *n* (%)	2 (1.8%)	4 (3.5%)	0.684
*EZH2*, *n* (%)	0 (0%)	2 (1.7%)	0.498
*SETBP1*, *n* (%)	0 (0%)	1 (0.9%)	1.000
*CBL*, *n* (%)	2 (1.8%)	3 (2.6%)	1.000
*PHF6*, *n* (%)	0 (0%)	2 (1.7%)	0.498
FAB, *n* (%)			0.113
M0	3 (2.8%)	10 (8.8%)	
M1	2 (1.9%)	9 (7.9%)	
M2	54 (50.5%)	51 (44.7%)	
M4	16 (15%)	14 (12.3%)	
M5	28 (26.2%)	28 (24.6%)	
M7	4 (3.7%)	2 (1.8%)

### High CSRP1 impacted the prognosis of AML

**Fig.3:**
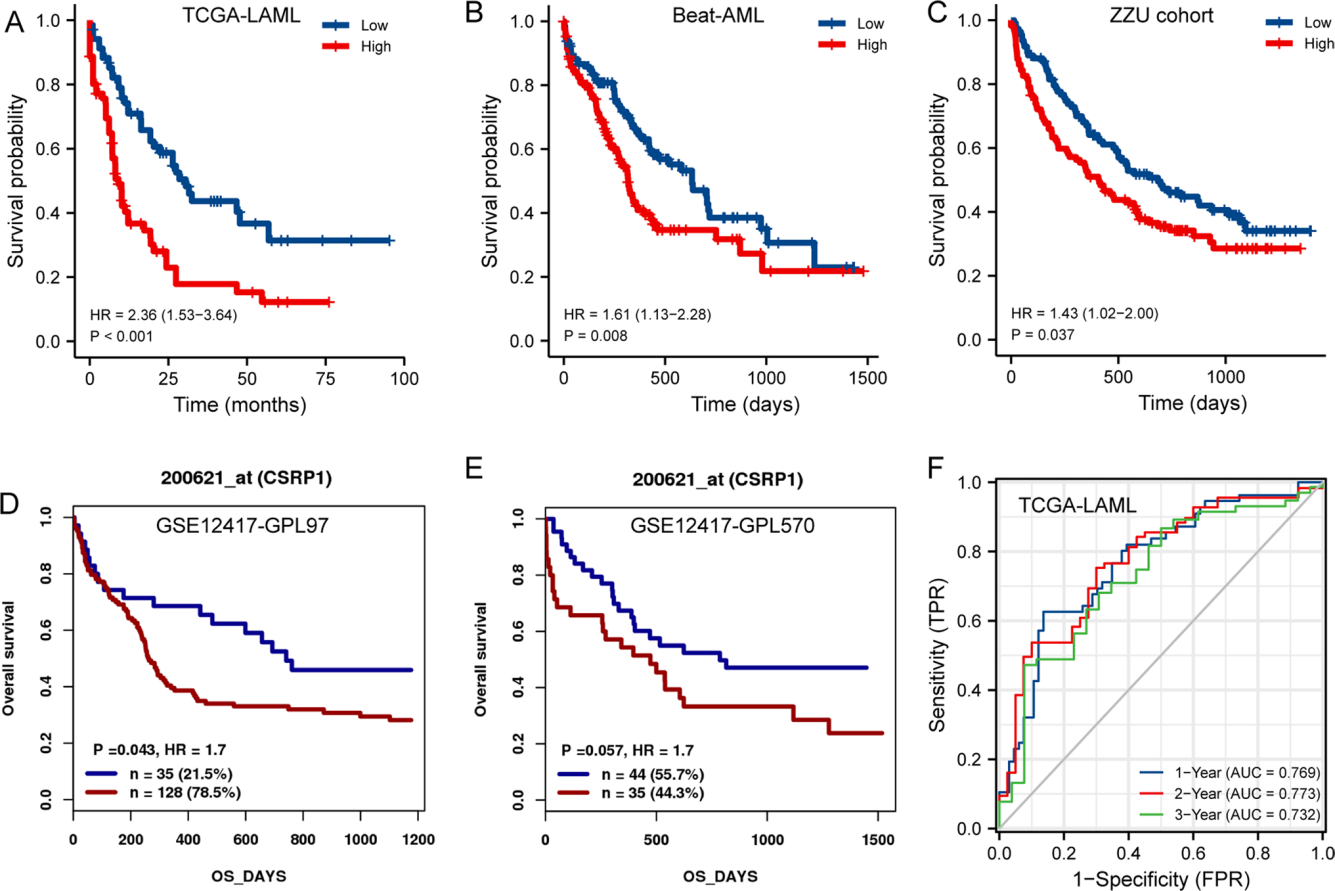
High expression of *CSRP1* was associated with poor OS in AML patients. **A**–**E** Kaplan–Meier curves of OS in the TCGA-LAML dataset **A**, the Beat-AML dataset **B**, the ZZU cohort **C**, the GSE12417-GPL96 dataset **D**, and the GSE12417-GPL570 dataset **E** and **F** Time-dependent ROC curve of *CSRP1* in TCGA-LAML dataset. *OS*, overall survival; *HR*, hazards ratio

Patients with high *CSRP1* expression had a significantly worse prognosis than those with low *CSRP1* expression (hazard ratio [*HR*], 2.36 (1.53–3.64); *P* < 0.001; Fig. 3A) in the TCGA-LAML dataset. The predictive significance of elevated *CSRP1* was verified in the Beat-AML dataset (Fig. 3B), the ZZU cohort (Fig. 3C), and the GSE12417 dataset (Fig. 3D–E). The time-dependent ROC curve from the TCGA-LAML dataset demonstrated that *CSRP1* was an excellent predictor of AML patient survival.

**Fig.4:**
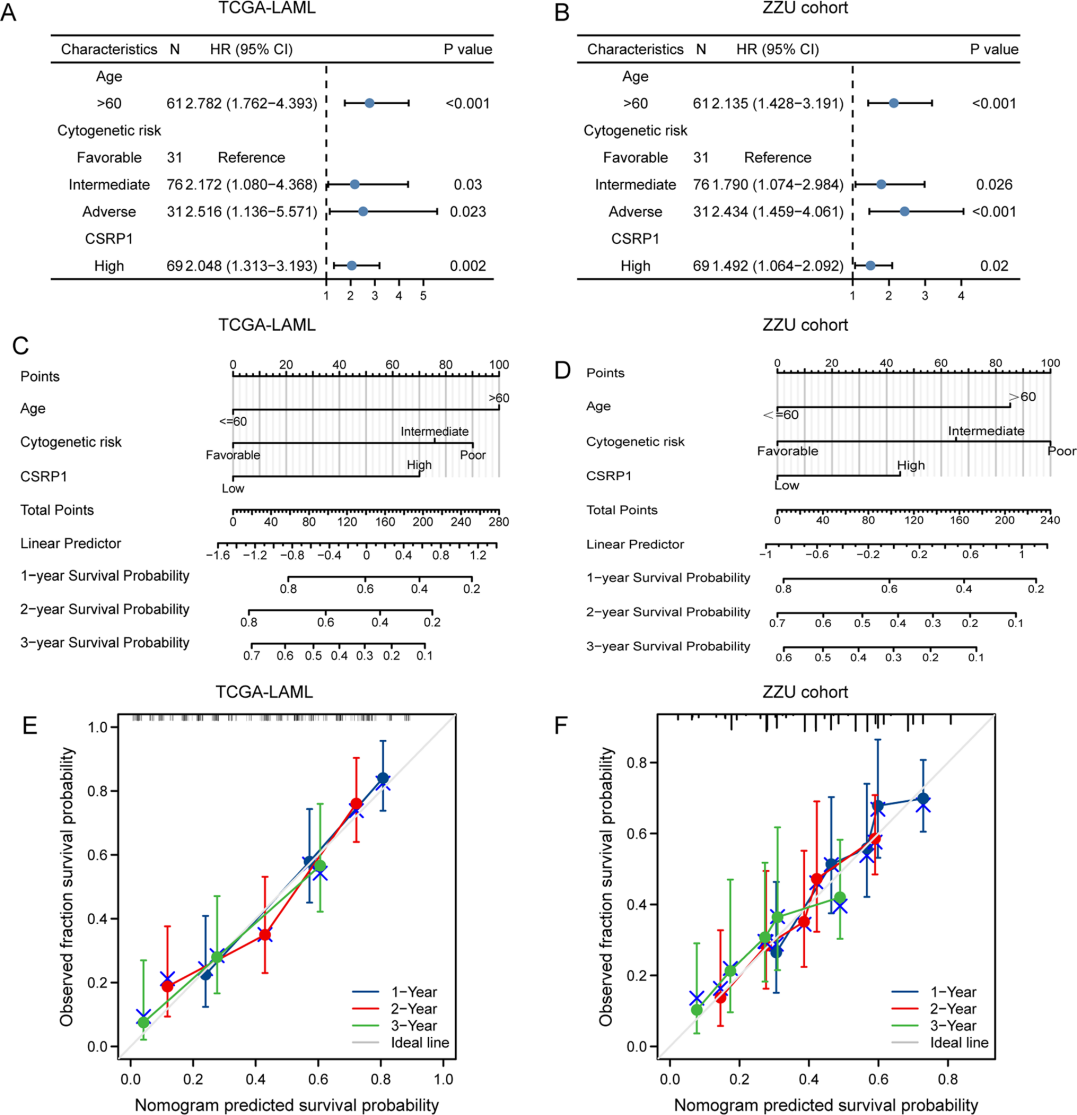
Construction of the nomogram model for AML patients. **A**–**B** The forest plots showed that age, cytogenetic risk, and *CSRP1* expression are independent factors for poor prognosis in the TCGA-LAML dataset **A** and the ZZU cohort **B**. **C**–**D** Nomogram for predicting the probability of 1-, 2-, 3-year OS for AML in the **C** TCGA-LAML dataset and **D** the ZZU cohort. **E**–**F** Calibration plot of the nomogram for predicting the probability of OS at 1, 2, and 3 years for AML in the **E** TCGA-LAML dataset and **F** the ZZU cohort

Based on the TCGA-LAML dataset, independent prognostic factors for worse OS included *CSRP1* (high vs*.* low, *P* < 0.001), cytogenetic risk (adverse vs*.* favorable, *P* < 0.001; intermediate vs*.* favorable, *P* = 0.002), and age (> 60 vs*.* ≤ 60, *P* < 0.001) (Table 2 and Fig. 4A). The independent prognostic value of these three factors was further confirmed in the ZZU cohort (Fig. 4B).

**Table 2:** Univariate analysis and multivariate analysis of the prognostic factors in the TCGA-LAML dataset

Characteristics	Univariate analysis		Multivariate analysis	
	*Hazard ratio* (95% *CI*)	*P* value	*Hazard ratio* (95% *CI*)	*P* value
Age	3.333 (2.164–5.134)	< 0.001	2.782 (1.762–4.393)	< 0.001
WBC	1.161 (0.760–1.772)	0.490		
Cytogenetic risk				
Favorable	Reference			
Intermediate	2.957 (1.498–5.836)	0.002	2.172 (1.080–4.368)	0.030
Adverse	4.157 (1.944–8.893)	< 0.001	2.516 (1.136–5.571)	0.023
*FLT3* mutation	1.271 (0.801–2.016)	0.309		
*NPM1* mutation	1.137 (0.706–1.832)	0.596		
BM-blasts(%)	1.165 (0.758–1.790)	0.486		
PB-blasts(%)	1.230 (0.806–1.878)	0.338		
*CSRP1*	2.356 (1.527–3.635)	< 0.001	2.048 (1.313–3.193)	0.002
Gender	1.030 (0.674–1.572)	0.892		

Age: > 60 versus <  = 60; WBC: > 20 × 10^ 9/L versus <  = 20 × 10^ 9/L; CSRP1: high CSRP1 expression versus low CSRP1 expression; Gender: male versus female

### Prognostic model of CSRP1 in AML

A nomogram was developed based on the Cox regression analyses in the TCGA dataset (Fig. 4C) and the ZZU cohort (Fig. 4D) to better predict AML patients' prognoses. Three independent prognostic variables, age, cytogenetic risk, and *CSRP1* expression, were included in the model at a statistical significance level of 0.05. A point scale was utilized to allocate points to these factors based on multivariate Cox analysis. Results from the nomogram calibration curve of OS prediction were consistent with observations of all patients in both the TCGA dataset (Fig. 4E) and the ZZU cohort (Fig. 4F).

### Identification of DEGs in AML samples with low and high CSRP1 expression

**Fig.5:**
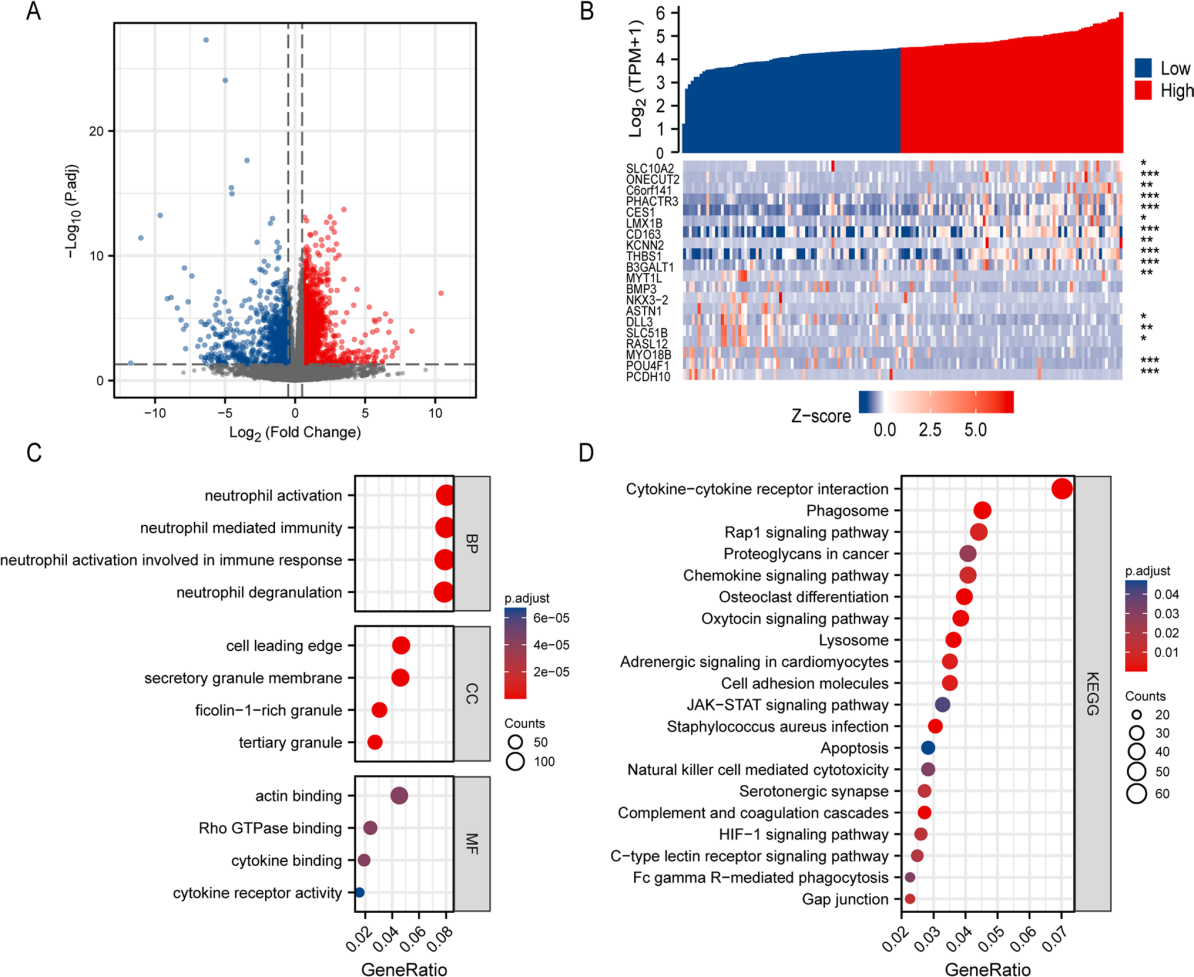
GO/KEGG enrichment analysis of DEGs comparing patients with high or low *CSRP1* expression in the TCGA-LAML dataset. **A** Volcano map of the DEGs, including 862 down-regulated genes and 1896 up-regulated genes. **B** Heat map showing the top ten up-regulated and the top ten down-regulated genes. The samples are shown on the X-axis, while the DEGs are shown on the Y-axis. **C**–**D** GO enrichment analysis of the up-regulated DEGs. MF, molecular function. CC, cellular component. BP, biological process. **D** KEGG enrichment analysis of the up-regulated DEGs. Different categories were shown on the Y-axis, while the X-axis reflected the percentage of DEGs

The gene expression profiles of the high and low *CSRP1* groups were analyzed for differences in median mRNA expression. A total of 2758 DEGs from RNA-seq-HTSeq counts were found to be statistically significant between the high and low *CSRP1* groups (|log fold change (logFC)|> 0.5, *P* adj < 0.05). (Fig. 5A). The heat map depicted the top ten up-regulated DEGs and top ten down-regulated DEGs between the high and low *CSRP1* groups (Fig. 5B).

### Functional enrichment analysis of DEGs

Biological process (BP) associated with high *CSRP1* included neutrophil activation, neutrophil activation involved in immune response, neutrophil degranulation, and neutrophil mediated immunity (Fig. 5C). Cellular components (CC) associated with high *CSRP1* included secretory granule membrane, ficolin-1-rich granule, tertiary granule, and cell leading edge (Fig. 5C). Molecular function (MF) associated with high *CSRP1* included actin binding, Rho GTPase binding, cytokine binding, and cytokine receptor activity (Fig. 5C). KEGG included phagosome, cytokine-cytokine receptor interaction, lysosome, oxytocin signaling pathway, cell adhesion molecules, rap1 signaling pathway, chemokine signaling pathway, gap junction, HIF-1 signaling pathway, proteoglycans in cancer, Fc gamma R-mediated phagocytosis, natural killer cell mediated cytotoxicity, JAK-STAT signaling pathway, and apoptosis (Fig. 5D).

**Fig.6:**
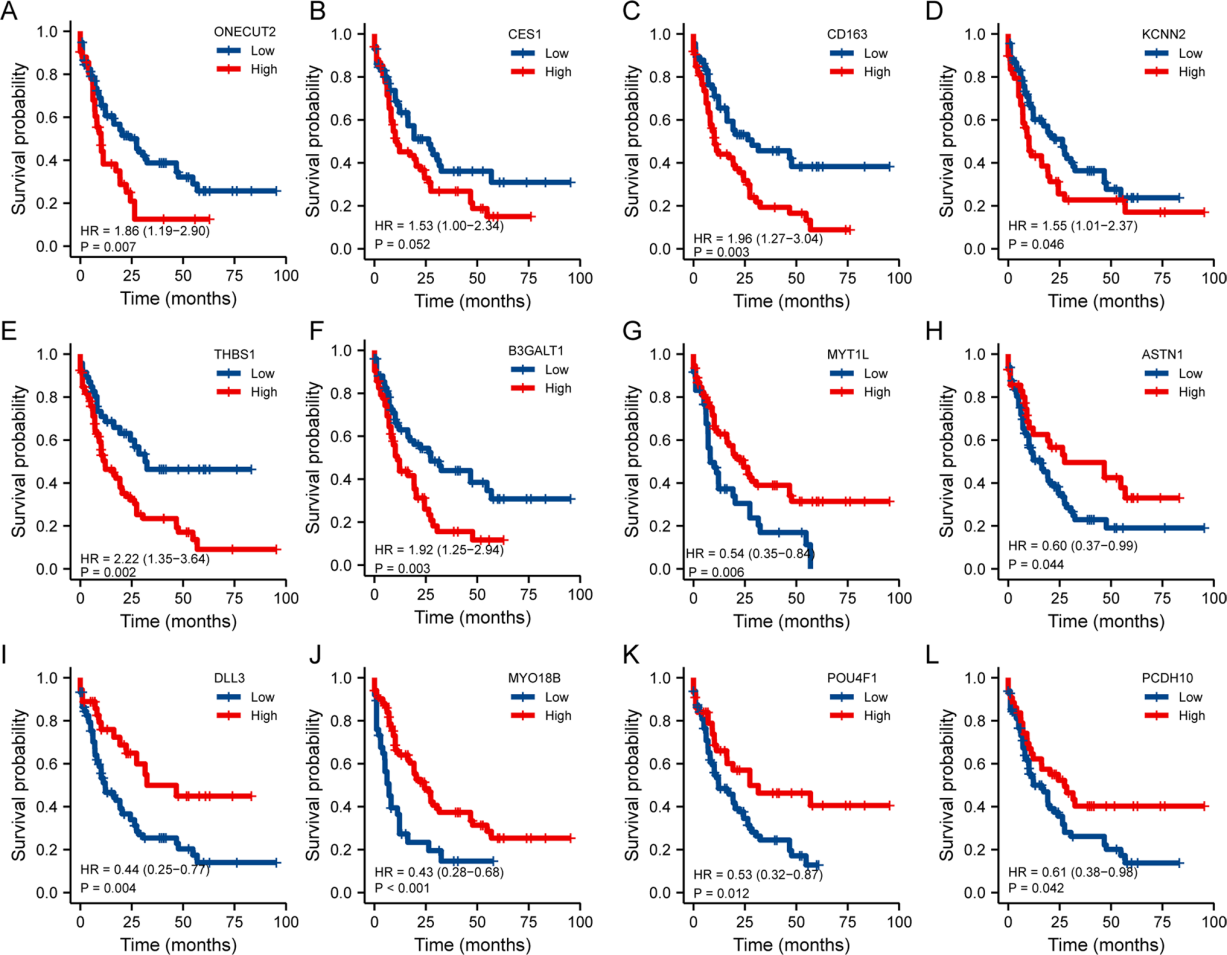
Prognostic value of DEGs in TCGA-LAML dataset. **A**–**F** Six of the top ten up-regulated DEGs (*ONECUT2* **A**, *CES1* **B**, *CD163* **C**, *KCNN2* **D**, *THBS1* **E**, and *B3GALT1* **F**) showed an adverse effect on OS. (**G**–**L**). Six of the top ten down-regulated DEGs (*MYT1L* **G** *ASTN1* **H**, *DLL3* **I**, *MYO18B* **J**, *POU4F1* **K**, and *PCDH10* **L**) had a protective effect on OS

The prognostic value of the top ten up-regulated DEGs and top ten down-regulated DEGs was further investigated. Six of the top ten DEGs that were up-regulated (*ONECUT2*, *CES1*, *CD163*, *KCNN2*, *THBS1*, and *B3GALT1*) and six of the top ten DEGs that were down-regulated (*MYT1L*, *ASTN1*, *DLL3*, *MYO18B*, *POU4F1*, and *PCDH10*) also had vital prognostic significance in the TCGA AML dataset (Fig. 6).

### Analysis of immune infiltration in AML

*CSRP1* expression in the AML microenvironment was associated with the level of immune cell infiltration as measured by ssGSEA. Mainly, *CSRP1* was negatively related to T cells and T helper cells and positively associated with macrophages (Fig.S1C), neutrophils (Fig.S1D), NK CD56 dim cells, NK CD56 bright cells, Tem, and iDC, TFH, Th17 cells, Th1 cells, eosinophils, Treg, and aDC (Fig.S1A). Patients with high *CSRP1* had higher enrichment scores of aDC, Eosinophils, iDC, macrophages, neutrophils, NK CD56 bright cells, NK CD56 dim cells, Tem, Th1 cells, Treg, and lower enrichment scores of T helper cells.

### PPI enrichment analysis in AML and validation of hub genes

The PPI network was constructed using the String website and the data were generated by Cytoscape (MCODE plug-in). A total of 321 DEGs were imported into the PPI network. Complete module including all genes is presented in Fig.S2A. We obtained 235 nodes and 900 edges. Nineteen genes were present in the most significant module (MCODE score: 7.667; Fig.S2B). The TCGA-LAML dataset was then used for OS analysis on the hub genes. Nine of the 19 hub genes, including *CD163*, *CX3CR1*, *C5AR1*, *THSD7A*, *ADMATS18*, *IL10*, *THBS1*, *ADAMTS15,* and *LILRB2*, were correlated with OS in AML (*P* < 0.05, Fig.S3).

### CSRP1 expression can predict sensitivity to the common chemotherapy agents in AML treatment

We utilized the “oncoPredict” tool to estimate sensitivity to frequently used chemotherapy agents to better correlate the *CSRP1* expression with clinical practice. Accordingly, drug sensitivity of patients in high and low *CSRP1* groups to multiple chemotherapy agents including 5-fluorouracil, gemcitabine, rapamycin, cisplatin, and fludarabine was predicted. Based on the findings, the high *CSRP1* groups of patients in the TCGA datasets showed higher sensitivity to 5-fluorouracil, gemcitabine, rapamycin, and cisplatin and lower sensitivity to fludarabine (Fig.S4). The relationship of sensitivity to other chemotherapy agents with *CSRP1* expression is listed in the supplementary data.

## Discussion

In this study, we explored the relationship between *CSRP1*, the clinicopathological features and prognosis of AML. We found that *CSRP1* was highly expressed in adult AML, which was associated with a higher proportion of bone marrow blasts, a higher frequency of DNMT3A mutation and a poor prognosis in AML patients. In addition, we constructed a nomogram to predict OS for AML based on age, cytogenetic risk stratification, and *CSRP1* expression levels and explored the possible mechanisms of *CSRP1* function.

Through pan-cancer analysis, we found that, unlike other tumor markers that are always highly or lowly expressed in different tumors, *CSRP1* shows different expression patterns between different tumors, with *CSRP1* highly expressed in 7 cancers and lowly expressed in 18 tumors. It suggests that *CSRP1* has a complex mechanism of regulation and can function as either an oncogene or oncogene suppressor in different cancer species or under different circumstances. In addition, the expression of *CSRP* gene family in AML is inconsistent, with the *CSRP1* highly expressed and *CSRP3* lowly expressed in AML. We have systematically investigated the role of the *CSRP2* gene in AML and found that its low expression is associated with poor prognosis in AML [15]. At the same time, the knockdown of *CSRP2* promotes proliferation and cycle progression in AML cell lines [15]. In contrast, no studies on *CSRP1* in AML have been reported. We confirmed the high expression of *CSRP1* in AML by comparing 224 adult AML patients with 23 healthy controls, which is consistent with the database results. All of these suggest that *CSRP1* may play an essential role in AML.

Next, we investigated the relationship between *CSRP1* gene expression, the clinicopathological features, and gene mutations using data from 224 adult AML cases. *CSRP1* expression did not correlate with gender, age, risk stratification, or WBC at diagnosis. The analysis of baseline data showed a higher proportion of bone marrow blasts and a higher frequency of *DNMT3A* mutations in the high *CSRP1* group. *DNMT3A* is one of the most frequently mutated genes in AML [31] and is an independent prognostic factor used for risk stratification [32]*.* It may suggest a higher tumor burden and a higher incidence of adverse prognostic mutations in those with high *CSRP1* expression.

We next explored the impact of *CSRP1* gene expression levels on overall survival in adult AML patients. We found that high *CSRP1* expression was associated with poor OS through multiple adult AML database cohort studies, including TCGA-LAML, Beat-AML, and GEO databases. Moreover, we further validated this result with the ZZU cohort. To further optimize the current stratification system for AML and facilitate clinical application, we first performed a univariate analysis. Initial screening revealed that age > 60 years old, worse cytogenetic risk stratification and high *CSRP1* expression were independent poor prognostic factors. The prognostic significance of age and karyotype stratification is well established. To facilitate clinical application, we further developed a nomogram and applied a calibration plot to validate the model. This model performed well for both the TCGA-LAML dataset and the ZZU cohort. This finding is beneficial for further optimizing the stratification system, especially for some patients classified as intermediate risk according to the current stratification, who can now better evaluate transplantation or chemotherapy according to the current guideline recommendation. This stratification system can further score, stratify and guide the treatment selection.

The overexpression of *CSRP1* and its associated poor prognostic value in AML suggest that it may play a role in AML. To further explore the mechanism of action of *CSRP1*, we subjected patients with high and low *CSRP1* expression to differential gene expression analysis, followed by GO and KEGG functional enrichment. GO-BP enrichment analysis revealed that *CSRP1* was closely associated with neutrophil function, which was confirmed in subsequent microenvironmental correlation analysis. Upregulating neutrophil elastase (NE) promoted the growth of leukemia cells and decreased the proportion of apoptotic cells [33]. GO-BP enrichment analysis reveals that *CSRP1* is associated with actin binding and Rho GTPs binding. AML with F*LT3-ITD* mutations is characterized by RAC1-dependent actin cytoskeleton remodeling that substantially contributes to the acquisition of resistance to midostaurin in vitro [34]. Yang et al. [35] analyzed the expression patterns and prognostic significance of Rho family GTPases in AML and found that *RhoBTB3* was significantly downregulated in AML bone marrow compared to healthy controls and correlated with prognosis of AML. KEGG enrichment analysis revealed that *CSRP1* was associated with cell adhesion molecules, rap1 signaling pathway, HIF-1 signaling pathway, JAK-STAT signaling pathway, and apoptosis. Cellular adhesion molecules also impact the poor prognosis of AML and may be used as targets for AML-specific therapies [36]. Also, the Rap1 signaling pathway plays a crucial role in cancer [37]. HIF signaling has been implicated in myeloid cell survival, and PI3K/Akt is known to induce HIF-1 transcription [38]. The signaling pathway JAK/STAT plays a critical role in the development and progression of AML [39].

In the PPI analysis, nine out of the 19 hub genes (*CD163*, *CX3CR1*, *C5AR1*, *THSD7A*, *ADMATS18*, *IL10*, *THBS1*, *ADAMTS15,* and *LILRB2*) were correlated with OS in AML. Notably, *CD163* and *THBS1* were among the 20 genes screened for the most significant differences based on *CSRP1* expression levels. *CD163* was a specific marker for macrophages of M2 type and was identified as a potential target for the therapeutic intervention of AML [40, 41]. The association of *CSRP1* with poor prognosis and macrophages in AML may be acting through *CD163*, but this requires further experimental confirmation. *THBS1* is a novel serum prognostic factors of AML [42]. *CX3CR1* was identified as one of the distinctive features of AML cells for universal MRD monitoring [43]. For AML patients who are ineligible for standard treatment with chemotherapy and HSCT and who also experience less severe side effects, Laura Jimbu et al. propose that manipulation of both the co-inhibitory network (with anti-PD-L1 blocking antibodies) and suppressor network (with anti-IL-10 blocking antibodies) is an appealing immunotherapeutic intervention. To increase the therapeutic effectiveness of treating AML illness, the suggested combination of these two immunotherapies provides a novel strategy that can be easily used in the clinic [44]. Endothelial cells (ECs)-derived small extracellular vesicles contained a high level of *ANGPTL2*, which accelerated leukemia progression via binding to the *LILRB2* receptor [45]. The other genes, including *C5AR1*, *THSD7A*, *ADAMTS18*, and *ADAMTS15*, have not yet been reported in AML-related studies and also require further investigation.

Finally, we also aimed to investigate whether *CSRP1* expression is capable of prognosticating the patients’ response to the commonly used chemotherapeutic agents. Our findings revealed that patients with higher *CSRP1* expression were more sensitive to certain chemotherapy agents including 5-fluorouracil, gemcitabine, rapamycin, and cisplatin and more resistant to fludarabine. This will help us in the selection of drugs in case of relapse and refractory patients.

In summary, *CSRP1* was highly expressed in adult AML, and such high-level expression of *CSRP1* was related to a poor prognosis in adult AML. *CSRP1* may serve as a potential prognostic marker and a therapeutic target for AML in the future. Further verification is expected to be carried out to reveal the clinical significance and biological impacts of *CSRP1* in AML.

## Supplementary Information

Below is the link to the electronic supplementary material.

Supplementary file1 (DOCX 1966 KB)

## Data Availability

The datasets generated and analyzed during the current study are not publicly available due to patient privacy considerations, but are available from the corresponding author on reasonable request (Shujuan Wang: fccwangsj1@zzu.edu.cn).
