# Supplementary material for: Cysteine- and glycine-rich protein 1 predicts prognosis and therapy response in patients with acute myeloid leukemia
Source: Clin Exp Med. 2024 Mar 28;24(1):57. doi: 10.1007/s10238-023-01269-w (PMC10978675; doi:10.1007/s10238-023-01269-w)
Supplement: Supplementary file 1 — Supplementary file1 (DOCX 1966 KB) [file 10238_2023_1269_MOESM1_ESM.docx]

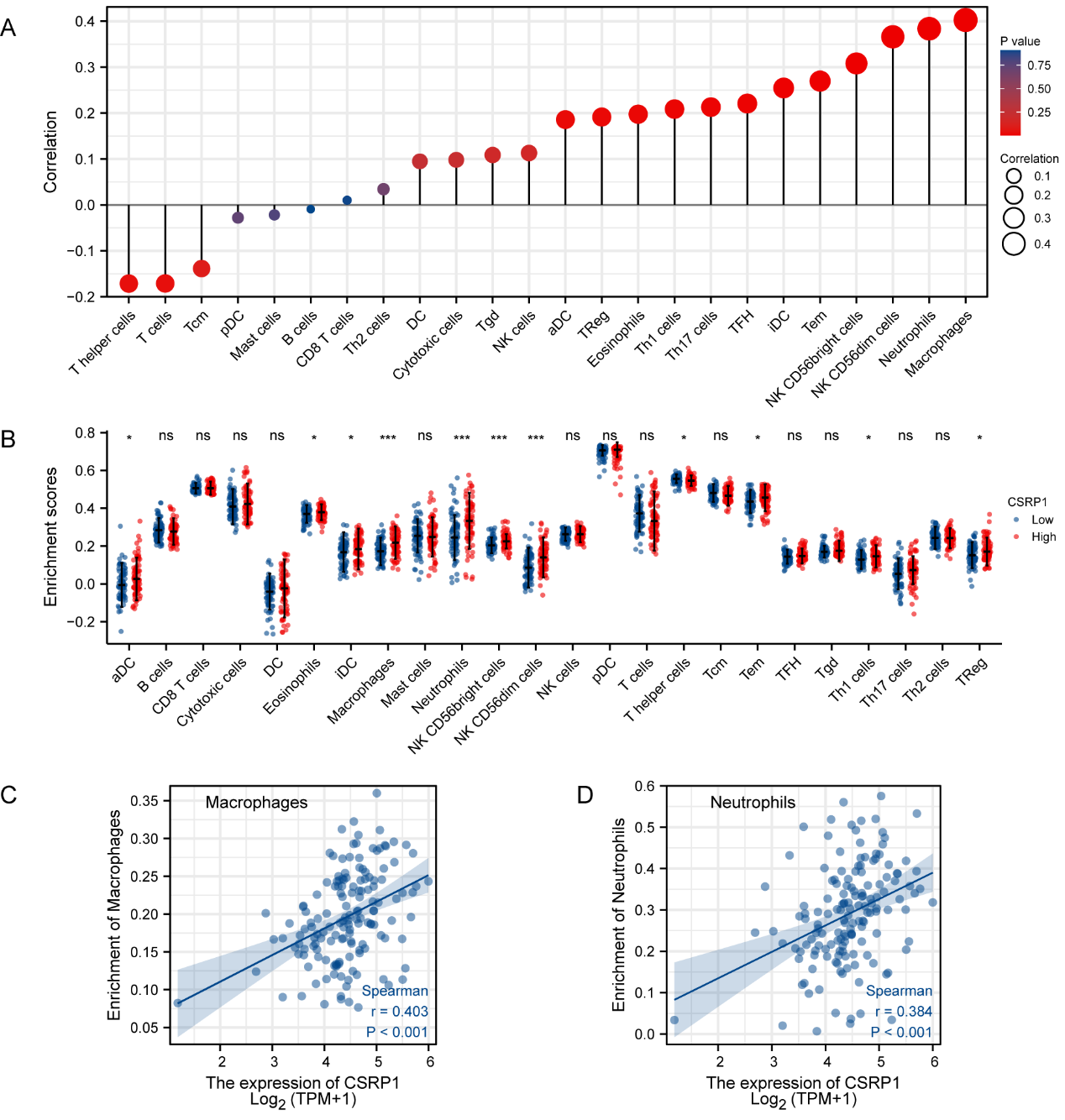


**Figure S1.** The expression of *CSRP1* was associated with immune infiltration in the AML microenvironment in the TCGA-LAML dataset. (A) The forest plots showed a positive correlation between *CSRP1* and 12 immune cells (macrophages, neutrophils, NK CD56 dim cells, NK CD56 bright cells, Tem, iDC, TFH, Th17 cells, Th1 cells, eosinophils, Treg, and aDC) and a negative correlation between *CSRP1* and two immune cells (T cells and T helper cells). The size of dots showed the absolute value of Spearman r. (B) The enrichment scores of different immune cell subsets in AML patients with low- and high- *CSRP1*. (C-D) Correlation between the expression level of *CSRP1* and the relative enrichment score of macrophages (C) and neutrophils (D). ns: *P* > 0.05; *, *P* < 0.05; **, *P* < 0.01; ***, *P*< 0.001.


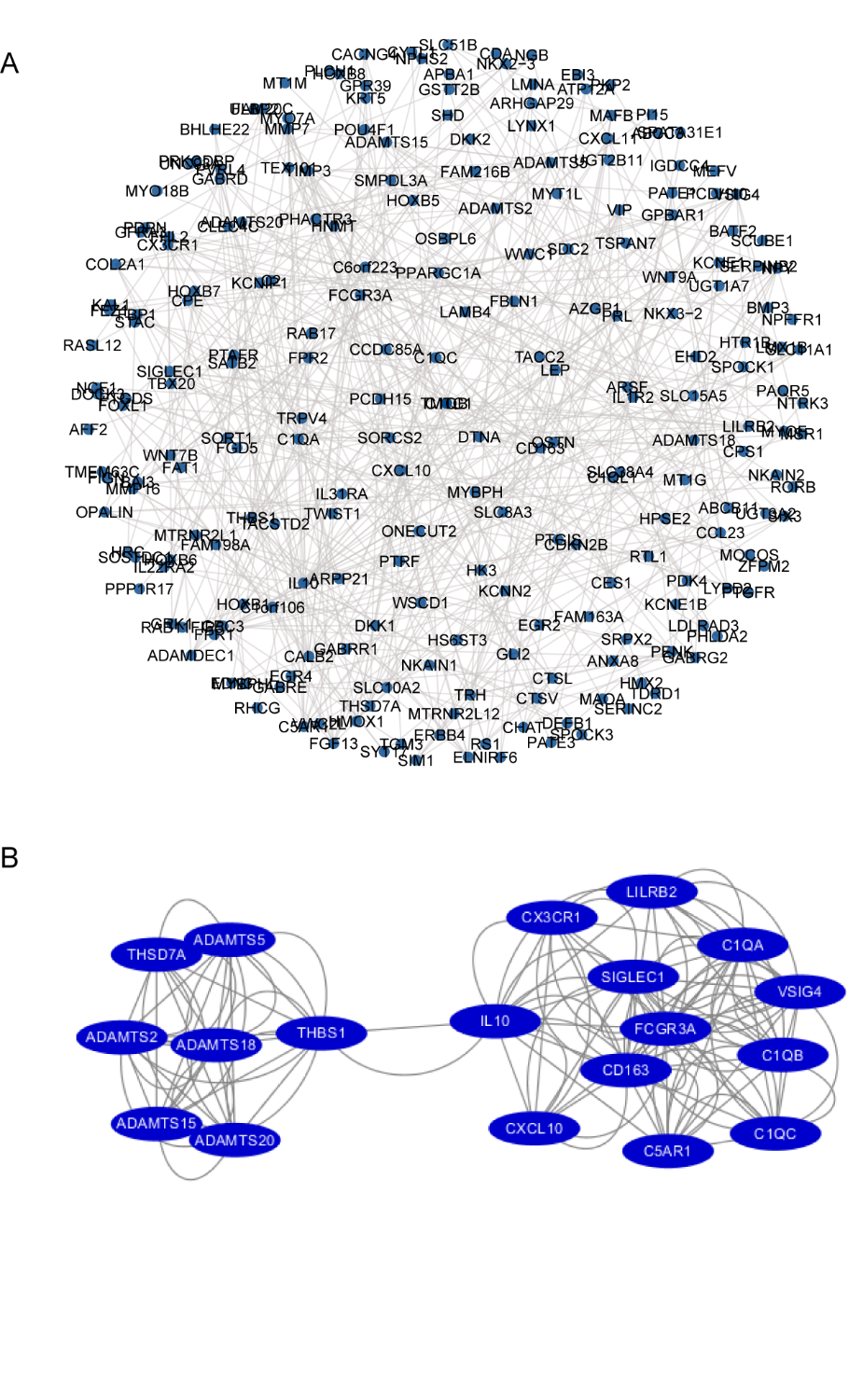


**Figure S2.** The PPI network of *CSRP1*-related DEGs and the most significant module. (A) Cytoscape was used to build the PPI network of DEGs. (B) The most significant module was obtained from PPI network with 19 nodes and 138 edges.


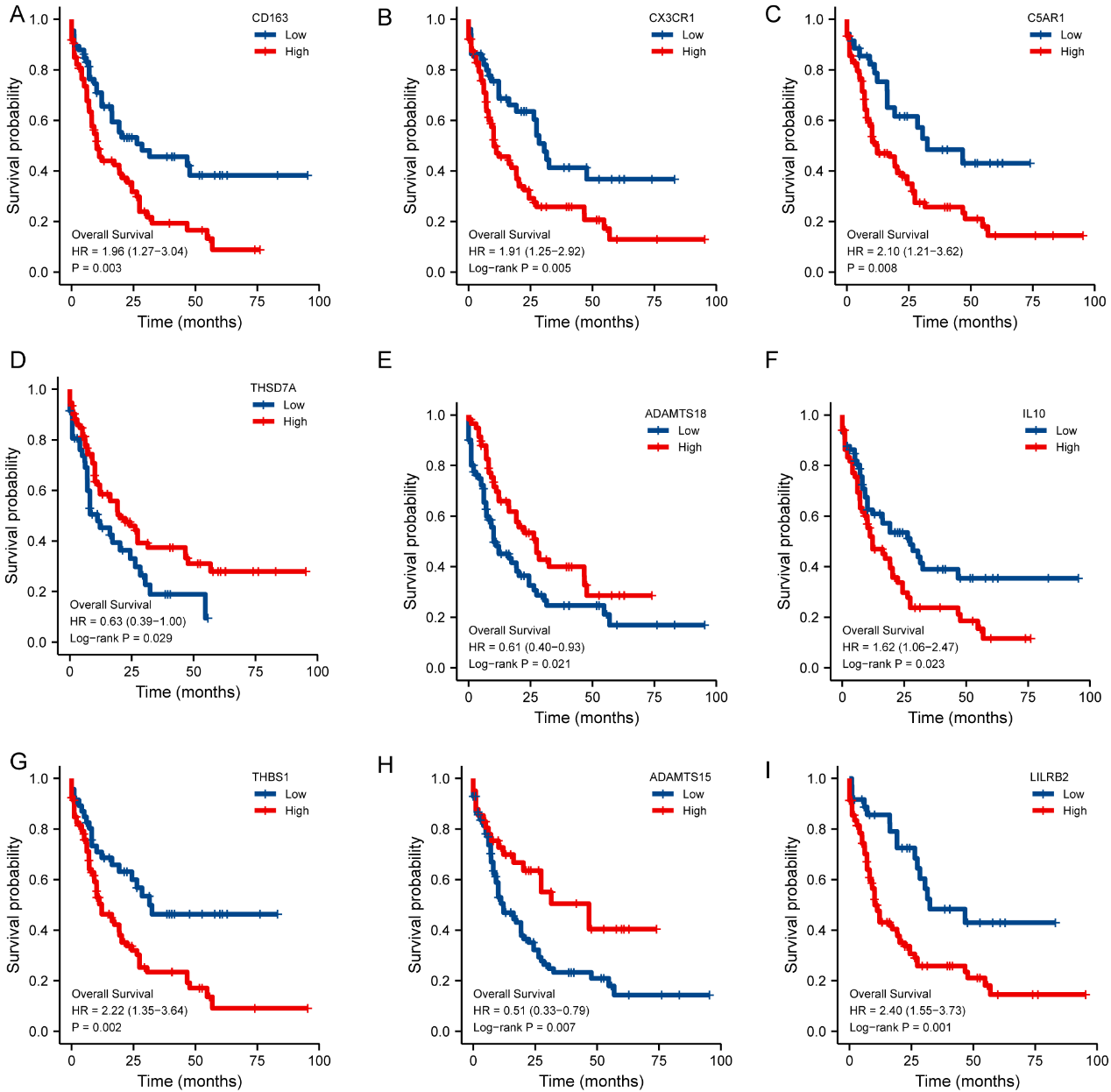


**Figure S3.** Prognostic values of the hub genes in TCGA-LAML cohort. (A) *CD163*, (B) *CX3CR1*, (C) *C5AR1*, (D) *THSD7A*, (E) *ADMATS18*, (F) *IL10*, (G) *THBS1*, (H) *ADAMTS15* and (I) *LILRB2*.


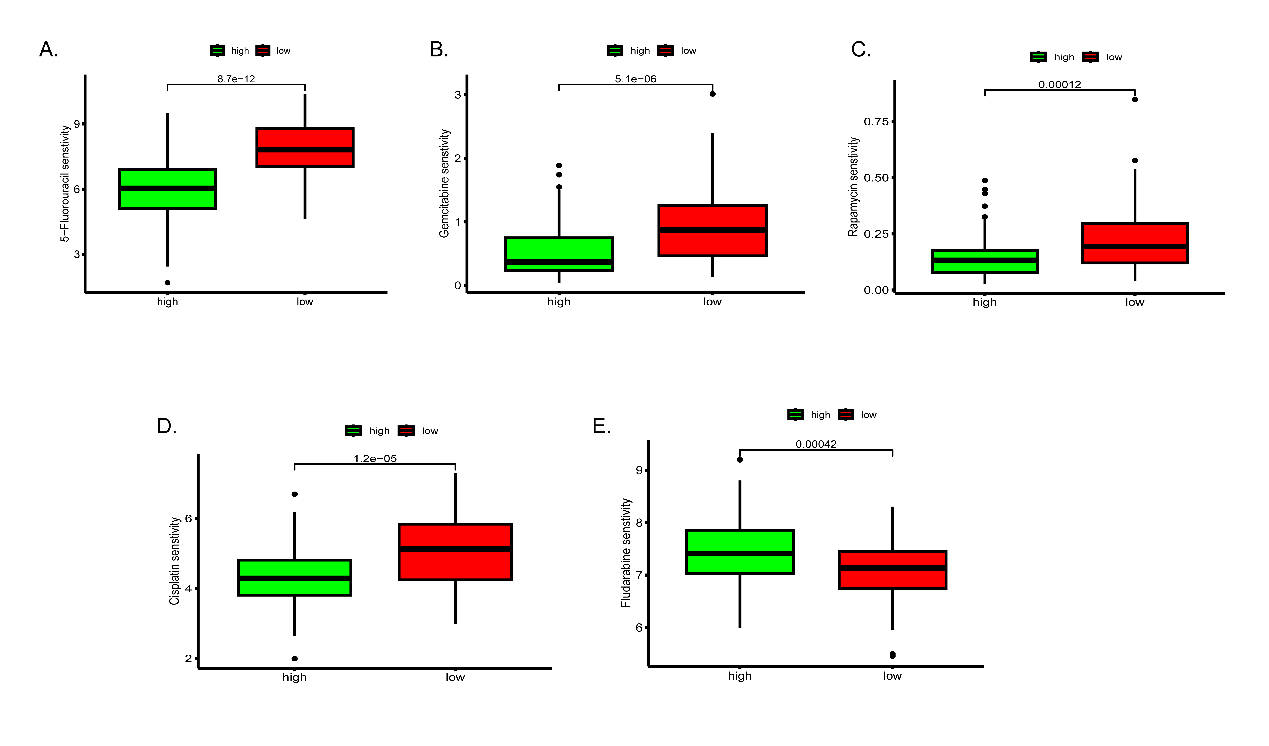


**Figure S4.** *CSRP1* expression was associated with chemotherapy response. The drug sensitivity of the high- and low-*CSRP1* patients to several chemotherapy agents 5-fuorouracil, gemcitabine, rapamycin, cisplatin and fludarabine was predicted using AUCs generated by OncoPredict. Lower AUC values represent higher sensitivities.

**Table S1 Sequences of primers and probes used in this study.**

| Name | Sequence (5'-3') |
| --- | --- |
| *CSRP1*- Forward primer | GAGGTTCAGTGCGAAGGCAA |
| *CSRP1*- Reverse primer  *CSRP1*- Probe | GCCTTTGGGCCCATACTTCT  FAM- ACAGTACCACTGTGGCCGTGCATGGT -BHQ |
| *ABL1*- Forward primer | TGGAGATAACACTCTAAGCATAACTAAAGGT |
| *ABL1*- Reverse primer  *ABL1*- Probe | GATGTAGTTGCTTGGGACCCA  FAM- CCATTTTTGGTTTGGGCTTCACACCATT -TAMARA |
